# Spinal Health during Unloading and Reloading Associated with Spaceflight

**DOI:** 10.3389/fphys.2017.01126

**Published:** 2018-01-18

**Authors:** David A. Green, Jonathan P. R. Scott

**Affiliations:** ^1^KBRwyle GmbH, Cologne, Germany; ^2^Space Medicine Office, European Astronaut Centre, European Space Agency, Cologne, Germany; ^3^Centre of Human and Aerospace Physiological Sciences, King's College London, London, United Kingdom

**Keywords:** back pain, spine, microgravity, axial loading, countermeasures, IVD herniation risk

## Abstract

Spinal elongation and back pain are recognized effects of exposure to microgravity, however, spinal health has received relatively little attention. This changed with the report of an increased risk of post-flight intervertebral disc (IVD) herniation and subsequent identification of spinal pathophysiology in some astronauts post-flight. Ground-based analogs, particularly bed rest, suggest that a loss of spinal curvature and IVD swelling may be factors contributing to unloading-induced spinal elongation. In flight, trunk muscle atrophy, in particular *multifidus*, may precipitate lumbar curvature loss and reduced spinal stability, but in-flight (ultrasound) and pre- and post-flight (MRI) imaging have yet to detect significant IVD changes. Current International Space Station missions involve short periods of moderate-to-high spinal (axial) loading during running and resistance exercise, superimposed upon a background of prolonged unloading (microgravity). Axial loading acting on a dysfunctional spine, weakened by anatomical changes and local muscle atrophy, might increase the risk of damage/injury. Alternatively, regular loading may be beneficial. Spinal pathology has been identified in-flight, but there are few contemporary reports of in-flight back injury and no recent studies of post-flight back injury incidence. Accurate routine in-flight stature measurements, in- and post-flight imaging, and tracking of pain and injury (herniation) for at least 2 years post-flight is thus warranted. These should be complemented by ground-based studies, in particular hyper buoyancy floatation (HBF) a novel analog of spinal unloading, in order to elucidate the mechanisms and risk of spinal injury, and to evaluate countermeasures for exploration where injury could be mission critical.

## Introduction

Insertion into microgravity (μG) is associated with fluid redistribution (Norsk et al., 2015), space adaptation syndrome (Thornton and Bonato, 2013) and increases in stature of up to 7 cm (Brown, 1977; Thornton et al., 1977) or 1–3% (Stoycos and Klute, 1993). Such increases are in excess of those (1% or 1–1.5 cm) observed after 8 h sleep on Earth (Tyrrell et al., 1985) and may be associated with back pain (Kerstman et al., 2012).

Increments in stature can present operational issues, such as astronauts being unable to fit into their extra vehicular activity (EVA) suit (e.g., NASA's EVA Mobility Unit; EMU; Nicogossian, 1989) that are assembled with a ~2.5 cm tolerance from pre-flight stature (Rajulu and Benson, 2009). Furthermore, presently all astronauts travel to the International Space Station (ISS) in the Russian Soyuz capsule, wearing the Sokol suit and fitted into a pre-molded “Kazbek” seat pan liner which, as a result of on-orbit stature increases, can be problematic.

Prior to EVA suit donning, stature is measured following the “On-orbit Growth Measurement protocol,” where an astronaut “stands” against a module wall whilst attempting to stabilize themselves by holding handrails whilst a second astronaut marks their height. In the Shuttle era, stature changes were assessed from seated height (Young and Rajulu, 2011) prior to re-entry (Nicogossian, 1977; Thornton and Moore, 1987). Except before an EVA, stature is no longer routinely measured on the ISS. However, evidence of spinal column changes (e.g., Chang et al., 2016) and a potential increased post-flight intervertebral disc injury risk (Johnston et al., 2010) has created a renewed interest in the effect of the space environment on spinal health. Due to the complexity of conducting human ISS experiments the majority of space spinal research is limited to pre- and post-flight which must be complemented by ground-based μG analogs.

## Ground-based analogs of microgravity for spinal research

The most commonly used ground-based analog of μG is long-duration, head-down tilt bed rest (HDTBR). Whilst HDTBR has significant utility in evaluating the effect of disuse and thus countermeasures for musculoskeletal de-conditioning (e.g., Rittweger et al., 2005), it is neither a true representation of the space gravitational environment [i.e., Earth's gravity acts “chest-to-back” (+Gx)] with a headward hydrostatic pressure gradient, nor is reflective of the ISS operational environment. Furthermore, up to 15 mins/day can be spent out of the head-down position, and spinal flexion (up to 30°), plus twisting and turning is permitted when head-down. This may explain why spinal elongation induced after 3 days of HDTBR is no greater than with 8 h sleep on Earth (Styf et al., 1997). It may also explain why both cervical muscle hypertrophy and thoracic intervertebral disc (IVD) compression is observed (Belavý et al., 2013) leading to questioning of its validity as an analog for spaceflight-induced spinal changes (Hargens and Vico, 2016).

Dry immersion, where individuals “float” in a partially “flexed” posture on an impermeable membrane via water buoyancy (Navasiolava et al., 2011) has also been used (Watenpaugh, 2016). Dry immersion induces significant back pain after only 1 day, but spinal elongation is moderate (1.5 cm) (Treffel et al., 2017). Furthermore, dry immersion is poorly tolerated, and in addition the head is supported out of the water which may result in cervical loading and neck afferent activation.

A novel ground-based analog of spinal unloading has been developed at King's College London, termed hyper-buoyancy flotation (HBF) (Green et al., 2015). In HBF, subjects lay supine upon a water bed encased within a frame, partially filled (50%) with a super-saturated and hence dense salt (magnesium sulfate) solution. Thus, subjects are buoyant, sinking into the bed in proportion to segmental body mass in a passive relaxed, supine position (negating the hydrostatic pressure gradient) with little or no requirement for stabilizing muscle activation. Four hours of HBF induces a stature increase of 1.8 ± 0.2 cm (Carvil et al., 2017b), comparable to that with 8 h of normal sleep (Tyrrell et al., 1985). However, greater elongation is observed after 8 h (2.4 ± 0.1 cm), as well as the development of moderate, reversible lower back pain that presents after 5–6 h (Green et al., 2015).

## Back pain: findings from microgravity and ground-based analogs

In fact, back pain features frequently in astronaut memoirs, with one stating that it “comes with the job” (Mullane, 2006) with back pain incidence ranging from 52 to 68% (Wing et al., 1991; Kerstman et al., 2012; Pool-Goudzwaard et al., 2015). Back pain was the fifth most common reason given for medication use in the Shuttle era (Putcha et al., 1999) and remains an issue on the ISS (Wotring, 2015). Lumbar pain is predominantly reported, typically presenting shortly after insertion into μG. Severity is most commonly reported as mild-to-moderate, although 25% is moderate-to-severe (Wing et al., 1991). Pain typically resolves after 2–3 days, although it can persist for more than a week. Astronauts are reported to adopt a “foetal” tuck in an attempt to reduce pain (Thornton et al., 1977), but this may risk spinal damage (Sayson et al., 2013). Whilst moderate back pain has also been reported after HDTBR (Hutchinson et al., 1995), dry immersion (Treffel et al., 2017), and HBF (Green et al., 2015), the underlying mechanisms remain unclear, although spinal lengthening may exaggerate intrathecal ligament tension (Kershner and Binhammer, 2004).

## Mechanisms underlying spinal changes and back pain with unloading

Intervertebral disc expansion, and spinal thoracic and lumbar curvature flattening have been proposed to explain μG-induced statue increases (Young and Rajulu, 2011). Loss of spinal curvature and IVD swelling have been observed post-HDTBR (Belavy et al., 2011a,b) in excess of that following 8 h' sleep (Ledsome et al., 1996). 60-day HDTBR has also been shown to reduce lumbar IVD signal intensity, indicative of reduced glycosaminoglycans concentration (Kordi et al., 2015). Three-day dry immersion is associated with increased lumbar IVD volume and water content (LeBlanc et al., 1994; Treffel et al., 2016), whereas the effect of HBF on IVDs is currently being evaluated.

Disc unloading is a critical feature of the daily IVD load-unload cycle, regulating composition and structure (Malko et al., 2002). As IVDs are largely avascular, and thus dependent upon membrane diffusion (Holm et al., 1981), the cycle promotes fluid/molecular exchange (Schmidt et al., 2016). Thus, IVD swelling is hypothesized to reduce diffusion and modify both osmotic and hydrostatic pressures (Humzah and Soames, 1988). Indeed, reduced protoglyocan and annuli fibrosus collagen (markers of disc degeneration) has been observed in rodent hindlimb suspension (Holguin and Judex, 2010) and μG (Maynard, 1994; Jin et al., 2013), although this remains to be confirmed in humans (Belavy et al., 2016a).

Utilisation of a new in-flight ultrasound procedure (Marshburn et al., 2014) with seven long-duration ISS astronauts, revealed 14 spinal changes from pre-flight, including disk desiccation and osteophytes, but no significant changes in IVD height or angle (Garcia et al., 2017). A recent MRI study (Chang et al., 2016) also suggests lumbar IVD swelling is minimal and comparable with that from an 8-day Shuttle mission (LeBlanc et al., 1994). However, post-flight images were recorded 24 and 48 h after landing, following exposure to Gz during re-entry and re-ambulation. Supine MRI images in the same astronauts showed decreased (11%) lumbar lordosis, and active lumbar flexion-extension range of motion (ROMFE) (Bailey et al., 2018), which are associated with impaired spine biomechanics and chronic low back pain on Earth (Hides et al., 1996; Freeman et al., 2010). In contrast, IVD water content and passive range of motion were unaffected. Thus, reduced lumbar lordosis may be a significant factor in spinal elongation, back pain, and potential herniation risk. However, the Bailey et al. (2018) study possessed no lumbar curvature, stature or back-pain in-flight measures, although only astronauts with significant pre-flight endplate irregularities (taskforce: Fardon et al., 2014) reported post-flight chronic low back pain and/or disc herniation (Bailey et al., 2018).

Back pain, the loss of lumbar lordosis and reduced ROMFE with μG exposure may be related to intrinsic (*rotatores, multifidus, semispinalis, spinalis, longissimus, iliocostalis)* spinal muscle atrophy, observed after 8 days (LeBlanc et al., 1995), and 17 days and 16–28 weeks (LeBlanc et al., 2000). Atrophy of the lumbar paraspinal muscles (*multifidus, erector spinae, quadratus lumborum*, and *psoas* at the level of L3/4 has recently been observed in NASA astronauts returning from ISS (Chang et al., 2016), consistent with routine ultrasonic observation of *lumbar multifidus* and *transversus abdominis* in European Space Agency (ESA) astronauts (Hides et al., 2016). *Multifidus* contributes to active sagittal and frontal plane stiffness (Panjabi et al., 1989), proprioception (Brumagne et al., 2008), and supports lordosis (Claus et al., 2009), consistent with limiting the forces acting upon IVDs and facet joints (Adams and Hutton, 1985) during bipedal gait (Sparrey et al., 2014).

Greater increases in *multifidus* signal intensity post-HDTBR (i.e., recovery) are associated with loss of lumbar (L4/L5) lordosis and the incidence of back pain (Belavy et al., 2011b). In astronauts, pre-post-flight changes in *multifidus* and *erector spinae* functional and anatomical cross-sectional area correlate with lordosis and active ROMFE (Bailey et al., 2018). Comparable trunk muscle atrophy has been seen after 60 days of HDTBR (Miokovic et al., 2012). Post-flight *multifidus* atrophy appears to reduce spinal joint stabilization, increase stiffness (Bailey et al., 2018) and directly affect IVDs (Adams, 2015). Thus, spinal stability may be a factor in determining spinal elongation, back pain, and potentially risk of IVD herniation (Belavý et al., 2016b), which may be elevated post-flight (Johnston et al., 2010). As a result, the Functional Re-adaptive Exercise Device (FRED), which engages *multifidus* and *transversus abdominis* (Weber et al., 2017) and is suitable even for those with back pain (Winnard et al., 2017), is currently being evaluated by ESA for post-flight rehabilitation.

## Spinal loading in-flight

Whilst long duration ISS missions might be described as “*approximately* six *months of uninterrupted spinal unloading*,” the current in-flight exercise countermeasure programme followed by ESA (Petersen et al., 2016) and other ISS astronauts (Loehr et al., 2015) involves both resistance and aerobic exercise (cycle ergometry or treadmill running), 6 days per week. When running (~30 min per session) on the 2^nd^ generation treadmill (T2) in order to be comparable to running on Earth (Genc et al., 2010), astronauts are restrained by an “over-the-shoulder” body harness that typically provides up to 70–80% body weight (Petersen et al., 2016). In addition, resistance exercises, such as squat, deadlift, and heel-raise, are performed on the Advanced Resistive Exercise Device (ARED), which provides axial loading up to 272 kg. The loads used in these exercises are typically in excess of those on Earth to compensate for the fact that astronauts are not working against their own body weight.

As such, only the spine above the level of the shoulders may be considered to be unloaded for an entire space mission. Below this level, a more appropriate description might be “*short periods of moderate to very high loading superimposed on a background of spinal unloading*.” What effect brief loading periods have on the spine is, as yet, unknown (Somers et al., 2015). For instance, on Earth spinal length reduces rapidly with loading induced by standing (Tyrrell et al., 1985), weight-training (Bourne and Reilly, 1991), and running (Dowzer et al., 1998), with additional upper limb loading causing further shortening (Fowler et al., 2006). Whether this is the case for treadmill and resistance exercise in μG is unknown, although astronauts have been reported to perform squats with ARED in an attempt to reduce stature.

Provision of static, axial loading may be a potential countermeasure to spinal elongation and/or back pain in μG. The Russian Pingvin (Penguin) suit was developed as a musculoskeletal countermeasure by imposing axial loading (reported to be ~40 kg) from the shoulder to foot (Gz) via bungee cords tethered to a waist belt (Sevrin and Svertshek, 1991; Kozlovskaya et al., 1995, 2015). Anecdotal reports suggest that it can transiently reverse stature elongation, although, due to the unnaturally high shoulder loading and poor thermal conductivity (and thus discomfort and skin hygiene issues), the suit is poorly tolerated.

To address the Pengvin Suit's limitations, the Gravity Loading Countermeasure SkinSuit (GLCS) was conceived to produce “1 Gz” using elastic fibers to generate multi-stage tension (that accumulates according to the proportion of body mass) in the vertical axis toward the feet (Waldie and Newman, 2011). Following various prototypes, the Mk III GLCS was found to provide ~0.7 Gz (measured at the feet) and shown to be compatible with acute strength (Carvil et al., 2017a) and aerobic exercise (Attias et al., 2017). Following several critical design and material innovations, the Mk VI SkinSuit was developed by ESA's Space Medicine Office and King's College London to specifically address whether the modified SkinSuit could reduce in-flight spinal elongation, without being uncomfortable or interfering with nominal ISS spaceflight activities. Ground-based studies using the HBF analog show that the Mk VI SkinSuit which provides axial loading equivalent to 20% of bodyweight both attenuates (Green et al., 2015) and reverses induced spinal elongation (Carvil et al., 2017b).

Following successful parabolic flight tests, the Mk VI SkinSuit was also evaluated during ESA's 2015 short duration “IRISS” and, more recently, during the 2016–17 “PROXIMA” long duration mission, where it partially reversed an increase in stature using a novel inflight stature measurement procedure that showed good within-session repeatability. However, whether the SkinSuit induces stature reductions in flight via lumbar IVD compression and/or induction of lordosis, as is observed with brief 10–30% bodyweight loading (Neuschwander et al., 2010; Shymon et al., 2014), is unknown. This and the effect on intervertebral motion/laxity (Du Rose and Breen, 2016) is currently the subject of further HBF studies.

## Looking forward: spinal health on long-duration exploration missions

Questions remain concerning the significance of the post-flight spinal changes (Chang et al., 2016; Garcia et al., 2017; Bailey et al., 2018) and their relationship to the apparent increased risk of post-flight IVD herniation (Johnston et al., 2010). In addition, whether the in-flight loading currently experienced by astronauts is protective or provocative with respect to spinal health is unclear. Might regular, albeit brief, axial loading be in some way protective of the spine and serve to attenuate spinal muscle atrophy? Or might applying loads, particularly the large loads used in some resistance exercises, on a spine already weakened muscle atrophy and/or anatomical changes increase the risk of damage and injury?

Of the 44 herniations reported by Johnston et al. (2010), only one occurred in an ISS astronaut and only four following long duration missions (Skylab and Mir). Whilst this might suggest a reduced IVD herniation risk with current ISS operations compared with earlier missions, the study of Johnston et al. (2010), by covering the period April 1959 to December 2006, includes only the first 6 years of the ISS long duration missions. Since that time, several important changes have occurred. Firstly, compared with earlier, shorter missions, immediate post-landing ambulation and activity (and thus axial loading) is more carefully managed following ISS missions, which may positively influence the risk of post-flight injury (Johnston et al., 2010).

The second important change is the in-flight loading environment. From December 2000, ISS crew utilized the Treadmill with Vibration Isolation and Stabilisation (TVIS) and performed resistance exercise on the Interim Resistive Exercise Device (iRED) (Korth, 2015). However, because of technical issues, in the first 4–5 years, TVIS was frequently operated with reduced harness loading, whilst the maximum iRED resistance was just 136 kg. Therefore, stronger crew easily reached the maximum load resulting in exercise specialists prescribing one-legged exercises and significant increases in exercise volume (repetitions and sets; Loehr et al., 2015). Exercise was the most frequent (12 of 14) cause of musculoskeletal injuries in ISS astronauts between 2000 and 2006 (Scheuring et al., 2009). Of those 12 injuries, nine were to the back, with the majority involving muscle strains sustained using iRED. In comparison, only 2 of 17 documented injuries from all the Gemini, Shuttle and NASA/Mir missions involved the back/spine.

In 2008, the ARED was installed on the ISS, with the T2 treadmill one year later, facilitating provision of frequent and higher spinal loading during exercise. Heavy squats, which are now possible with ARED, may produce high “uncontrolled” instantaneous, impulse loads through the spine, and thus may present an injury risk (Jennings and Bagian, 1996; Scheuring et al., 2009), particularly as IVDs are relatively uncompliant (Maquer et al., 2014). However, there are no published in-flight injury data from 2006 onwards, during which time ARED and T2 have been fully operational. That said, since their introduction, no ESA astronaut has experienced a back injury resulting from in-flight exercise that has required modification of their countermeasure programme. Thus, whilst the effect of this enhanced loading environment on spinal health has yet to be determined, evidently spinal muscle atrophy and structural changes remain (Chang et al., 2016; Hides et al., 2016; Garcia et al., 2017; Bailey et al., 2018).

## Conclusion

Space-related spinal elongation, back pain, and elevated risk of IVD herniation have historically been considered to be related to IVD swelling. However, recent evidence suggests that trunk musculature atrophy, in particular *multifidus*, may precipitate loss of lumbar curvature (and thus stature increments) and lead to spinal instability and IVD dysfunction. Current ISS missions involve short periods of moderate-to-high axial loading, superimposed upon a background of prolonged unloading, yet it is unknown how these affect spinal health, and the risk of post-flight IVD herniation. As a result, routine accurate in-flight stature measurements combined with back pain recording should supplement spinal imaging, both in-flight, and as soon as possible upon landing coupled with extended spinal health tracking post-flight. In addition, evaluation of the effect of acute and repetitive graded axial loading is warranted both in orbit and in appropriate ground-based analogs such as HBF. Data from such investigations will help understand the role of unloading and in-flight loading upon spinal health, and therefore spinal injury risk, which could be critical in future human exploration missions.

## Author contributions

All authors listed have made a substantial, direct and intellectual contribution to the work, and approved it for publication.

### Conflict of interest statement

The authors declare that the research was conducted in the absence of any commercial or financial relationships that could be construed as a potential conflict of interest.

## Abbreviations

ARED: Advanced Resistive ExerciseDevice
Gz: Axial acceleration
ESA: European Space Agency
EMU: EVA Mobility Unit
EVA: Extra vehicular activity
FRED: Functional Re-adaptive Exercise Device
GLCS: Gravity Loading Countermeasure SkinSuit
HDTBR: Head-down tilt bed rest
HBF: Hyper-buoyancy floatation
iRED: Interim Resistive Exercise Device
ISS: International Space Station
IVD: Intervertebral disc
MRI: Magnetic reasonance imaging
μG: Microgravity
NASA: National Aeronautics and Space Administration
ROMFE: Range of motion - flexion-extension
TVIS: Treadmill with Vibration Isolation and Stabilisation
T2: 2^nd^ generation treadmill.
